# Platelet rich plasma injection for acute Achilles tendon rupture: PATH-2 randomised, placebo controlled, superiority trial

**DOI:** 10.1136/bmj.l6132

**Published:** 2019-11-20

**Authors:** David J Keene, Joseph Alsousou, Paul Harrison, Philippa Hulley, Susan Wagland, Scott R Parsons, Jacqueline Y Thompson, Heather M O’Connor, Michael Maia Schlüssel, Susan J Dutton, Sarah E Lamb, Keith Willett

**Affiliations:** 1 Kadoorie Research Centre, Nuffield Department of Orthopaedics, Rheumatology and Musculoskeletal Sciences, University of Oxford, Oxford, UK; 2 Institute of Translational Medicine, University of Liverpool, Liverpool, UK; 3 Institute of Inflammation and Ageing, University of Birmingham, Birmingham, UK; 4 Botnar Research Centre, Nuffield Department of Orthopaedics, Rheumatology and Musculoskeletal Sciences, University of Oxford, Oxford, UK; 5 Oxford Clinical Trials Research Unit, Centre for Statistics in Medicine, Nuffield Department of Orthopaedics, Rheumatology and Musculoskeletal Sciences, University of Oxford, Oxford, UK

## Abstract

**Objective:**

To determine whether an injection of platelet rich plasma improves outcomes after acute Achilles tendon rupture.

**Design:**

Randomised, placebo controlled, two arm, parallel group, participant and assessor masked, superiority trial.

**Setting:**

Secondary care trauma units across 19 hospitals in the United Kingdom’s health service.

**Participants:**

Recruitment commenced in July 2015 and follow-up was completed in March 2018. 230 adults aged 18 years and over were included, with acute Achilles tendon rupture presenting within 12 days of injury and managed with non-surgical treatment. Exclusions were injury at the insertion or musculotendinous junction, major leg injury or deformity, diabetes mellitus, platelet or haematological disorder, systemic corticosteroids, anticoagulation treatment, and other contraindicating conditions.

**Interventions:**

Participants were randomised 1:1 to platelet rich plasma (n=114) or placebo (dry needle; n=116) injection. All participants received standard rehabilitation care (ankle immobilisation followed by physiotherapy).

**Main outcomes and measures:**

Primary outcome was muscle tendon function at 24 weeks, measured objectively with the limb symmetry index (injured/uninjured×100) in maximal work done during the heel rise endurance test (an instrumented measure of repeated single leg heel rises until fatigue). Secondary outcomes included patient reported function (Achilles tendon rupture score), quality of life (short form 12 version 2®), pain (visual analogue scale), goal attainment (patient specific functional scale), and adverse events. A central laboratory analysed the quality and content of platelet rich plasma. Analyses were by modified intention to treat.

**Results:**

Participants were 46 years old on average, and 57 (25%) of 230 were female. At 24 weeks, 202 (88%) participants completed the heel rise endurance test and 216 (94%) the patient reported outcomes. The platelet rich plasma was of good quality, with expected growth factor content. No difference was detected in muscle tendon function between participants receiving platelet rich plasma injections and those receiving placebo injections (limb symmetry index, mean 34.7% (standard deviation 17.7%) *v* 38.5% (22.8%); adjusted mean difference −3.9% (95% confidence interval −10.5% to 2.7%)) or in any secondary outcomes or adverse event rates. Complier average causal effect analyses gave similar findings.

**Conclusions:**

There is no evidence to indicate that injections of platelet rich plasma can improve objective muscle tendon function, patient reported function, or quality of life after acute Achilles tendon rupture compared with placebo, or that they offer any patient benefit.

**Trial registration:**

ISRCTN54992179.

## Introduction

The most commonly ruptured tendon is the Achilles, and the incidence is rising.^1^ ^2^ After Achilles tendon rupture, patients experience limitations in tendon loading activities, resulting in work incapacity and many months off sport.^3^ ^4^ Accelerating recovery and improving quality of the tissue repair are therefore desirable.

Platelet rich plasma is an autologous, whole blood product that provides a supraphysiological concentration of platelets, leucocytes, growth factors, and other bioactive proteins such as cytokines and chemokines for delivery to an injury site.^5^ As platelet rich plasma has shown positive cellular and physiological effects on tendon healing under laboratory conditions in most studies,^6^ it is now used extensively in sports and musculoskeletal orthopaedic medicine, with the market projected to be worth US$383.56m (£297.20m; €344.49m) by 2023.^7^ Its popularity has been fuelled by much media attention following its use by elite athletes.^8^ However, commercial preparation systems of platelet rich plasma only have US Food and Drug Administration approval based on device performance and safety, not clinical efficacy.^9^ Despite 37 clinical trials on applications of platelet rich plasma in musculoskeletal injuries so far, its efficacy remains uncertain.^10^ ^11^ These trials, and the two previous trials of platelet rich plasma in Achilles rupture,^12^ ^13^ are hampered by a lack of standardisation of platelet rich plasma preparations and quality control, underpowered studies, and potential confounders such as concurrent surgery.^14^

The Achilles tendon rupture offers an optimal clinical model for determining the efficacy of platelet rich plasma because it has a relatively homogenous presentation, and is easy to diagnose and mechanically test, compared with other tendon rupture sites. We aimed to determine the clinical efficacy of a standardised preparation of platelet rich plasma in treating acute, non-surgically managed rupture of the Achilles tendon in a randomised controlled trial. We hypothesised that if platelet rich plasma accelerated tendon healing and improved mechanical properties of the healing tissue, it would result in improved muscle tendon function of participants.

## Methods

### Study design and eligibility criteria

PATH-2 was a randomised, placebo controlled, multicentre, two arm, parallel group, superiority trial with masked participants and outcome assessors conducted at 19 hospitals in the United Kingdom. The trial methods, interventions, and analysis plan have been published.^15^ ^16^

### Participants

We included adults who were aged 18 and over; had a clinical diagnosis (with or without confirmatory diagnostic imaging) of a complete acute mid-substance rupture of the Achilles tendon made by the treating clinician in the outpatient orthopaedic trauma clinic; were within 12 days of injury; were able to walk unaided pre-injury; and were being managed non-surgically by immobilising the ankle in a cast, splint, or boot. We excluded those individuals clinically diagnosed with a tendon rupture at the insertion or musculotendinous junction, previous major leg injury or deformity, diabetes mellitus, platelet or haematological disorder, current systemic corticosteroids, treatment doses of anticoagulation treatment, and other contraindicating conditions (lower limb gangrene/ulcers, peripheral vessel disease, hepatic or renal failure or dialysis, pregnant or breast feeding, radiation or chemotherapy in previous three months, inadequate venous access). 

In May 2016, nine months into recruitment, the eligibility criteria were amended (see supplementary file for details on all protocol amendments). A key change was an extension of the maximum number of days since injury, from seven to 12 days. During the initial months of screening, we noted that most patients were arriving at clinics within 12 days for acute treatment, which meant that many patients in the target population were not being offered entry to the study. All participants provided written informed consent.

### Randomisation

Following consent and baseline assessments, participants were individually randomised 1:1 to an injection of platelet rich plasma or placebo via a central 24 hour, web based, randomisation allocation system developed and provided by the Oxford Clinical Trials Research Unit. Initial randomisation was done in variable permuted blocks stratified by study site and age group (<55 *v* ≥55 years). However, the age groups had not been implemented, owing to a technical issue, which led to an imbalance of treatment across age groups. To provide balance at the end of the study over stratification factors, the system was changed to minimisation using the existing randomised participants, including a probabilistic element (0.8) to prevent predictability of treatment allocation. Minimisation factors were study site and age group as originally intended.^17^ These amendments were approved by the trial steering, data monitoring, and ethics committees.

### Interventions

Participants from both groups attended the centres within 12 days of injury to have blood withdrawn and receive an injection in the tendon gap during one visit. Participants randomly assigned to receive platelet rich plasma had 55 mL of venous blood withdrawn. From this volume, 5 mL was used for whole blood analysis and 50 mL was used to produce 8 mL of leucocyte and platelet rich plasma using the same model of specialised automated centrifuge (MAG 200 MAGELLAN Autologous Platelet Separator, Arteriocyte Medical Systems, MA) and sterile disposable kit (MDK 300/300-1, Arteriocyte Medical Systems, MA) in all centres. Participants in the placebo group still had 5 mL of venous blood withdrawn that was used for whole blood analysis. Participants from both groups waited for about 17 minutes after blood withdrawal before receiving the injection (time taken to prepare the platelet rich plasma).

For both interventions, participants lay face down on a treatment table. The treating surgeon or specialist physiotherapist palpated the tendon gap to identify the injection site. Use of imaging was not necessitated by the trial protocol. The clinician cleaned the site, injected a local anaesthetic (1-2 mL) into the skin, and then injected the intervention in the centre of the tendon gap. The platelet rich plasma group had 4 mL of platelet rich plasma injected. The remaining 4 mL of platelet rich plasma was processed for laboratory analysis and quality control. The placebo group had the same sized needle attached to an empty syringe inserted into the tendon gap, held in place for the duration of a platelet rich plasma injection, and withdrawn without injecting anything. All treating clinicians undertook study specific training, used a step-by-step preparation manual, and had access to a training video.

After injection, participants continued with usual local non-surgical care for a ruptured Achilles tendon including local protocols for venous thromboembolism prophylaxis, except that we standardised the rehabilitation protocol to reduce substantial variations between groups and recruiting hospitals. The ankle was initially immobilised in an equinus position for at least three weeks after the intervention. Clinicians were instructed to avoid participants’ having full time ankle immobilisation or non-weight bearing for longer than six weeks. All participants were referred to a physiotherapist for supervised rehabilitation. Adherence to rehabilitation protocol was monitored in the participant reported questionnaires at 4, 7, and 13 weeks.

The whole blood and platelet rich plasma samples were analysed in a central laboratory at the Institute of Inflammation and Ageing, University of Birmingham, Birmingham, UK. Whole blood and platelet rich plasma cell counts were determined by a Sysmex XN-1000 Haematology analyser (Sysmex UK, Milton Keynes, UK). The instrument provides three different platelet counts; impedance, optical, and fluorescent. Where possible, the fluorescent platelet count was the preferred platelet count used. Instrument performance was checked internally daily (XN Check) and externally monthly (UKNEQAS, Watford, UK)^18^ to ensure quality and accuracy. Platelet quality within fixed resting and activated samples (PAMfix, Platelet Solutions, Nottingham, UK) was analysed by measuring the expression of P selectin (CD62p), a platelet specific activation marker, by flow cytometry (Accuri Flow cytometer, Becton Dickinson, Oxford, UK). Growth factor concentrations (of platelet derived growth factor-AB, insulin-like growth factor 1, vascular endothelial growth factor, fibroblast growth factor-basic, and transforming growth factor β1) within the platelet rich plasma were measured by enzyme linked immunosorbent assay (see supplementary file for further sample preparation information).

### Masking

All participants were informed that up to 55 mL of venous blood would be taken, but the exact amounts for each intervention were not disclosed. It was agreed with the ethics committee that the protocol and patient information sheet would only contain the maximum volume of blood to be taken. In practice, differing volumes of blood were taken because it was deemed unacceptable to take more blood than required for study participation and treatment. Injections were prepared out of participants’ sight while they waited. A dummy spin cycle was activated for the placebo group if the platelet rich plasma centrifuge was within audible range. Participants received the injection lying face down with instructions not to turn to view the procedure or injection syringes. Primary outcome assessors were not aware of allocation or treatment. Clinicians involved in preparing or delivering the intervention could not be masked. We trained all staff involved in preparation and delivery and provided a comprehensive manual with step-by-step instructions to facilitate masking. It was emphasised that the intervention should not be discussed with participants.

### Outcomes and study assessments

The primary outcome time point was 24 weeks, when participants attended an appointment to complete self report questionnaires on secondary outcome measures and a clinical assessment. Follow-up questionnaires were also completed face-to-face or by telephone at 4, 7, and 13 weeks after randomisation by a research associate at the recruiting centre. Questionnaires were also sent in the post by research associates, or collected over the phone by a researcher in the clinical trials unit, to optimise follow-up.

**Fig 1:**
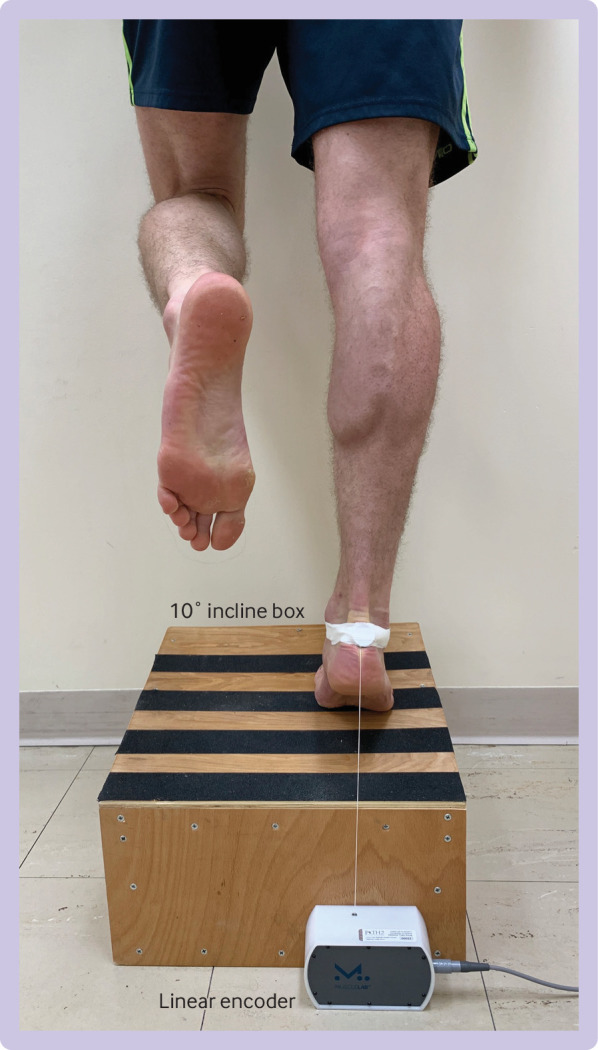
Participant performing the heel rise endurance test

The primary outcome was muscle tendon function as measured by the validated heel rise endurance test.^19^ Participants stood on each leg in turn and raised and lowered the heel until fatigued. A computer controlled linear encoder (MUSCLELAB, Ergotest Innovation, Porsgrunn, Norway) and video recordings collected the movement data. The linear encoder measured the height of each heel rise, which was used with body weight to calculate the work performed by each lower limb in joules. Performance was expressed as a limb symmetry index (injured limb with rupture/uninjured limb measurement×100) for the maximal work done during the heel rise endurance test. Two members of the study team, masked to treatment allocation, independently reviewed all the assessment videos and discounted any invalid recordings in the data from the heel rise endurance test (supplementary file and fig 1 provides further information on procedures for the heel rise endurance test).

Secondary outcomes were the number of heel rise repetitions and maximum heel rise height (cm) during the heel rise endurance test at 24 weeks after randomisation, patient reported symptoms and function (Achilles tendon rupture score 0-100, higher score better),^20^ functional limitation due to pain (Achilles tendon rupture score 0-10, higher score better; not specified in the protocol but planned before completion of follow-up and reported in the published statistical analysis plan),^16^ functional goal attainment (patient specific functional scale^21^ ^22^ 0-10, higher score better), and health related quality of life (short form 12 version 2® (v2) Health Survey, acute version, 0-100, higher score better)^23^ at 4, 7, and 13 weeks after randomisation. Participants assessed their pain in a daily diary using a visual analogue score (0-100, lower score better)^24^ for two weeks after the intervention.

Expected complications, harms, and further interventions related to the study treatments were recorded as adverse events. Serious adverse events were defined as any untoward medical occurrence that was both unexpected and related to the study treatments that resulted in: death within 30 days of the intervention, death related directly to the intervention at any time, life or limb threatening complication, or readmission to hospital. Treatment relatedness was determined by the site and confirmed by the chief investigator.

### Sample size

The initial sample size target was 214, based on detecting a standardised difference of 0.5 at 24 weeks in the primary outcome (based on data from a previous trial where a clinically important difference of 10% with a standard deviation of 20% from the non-surgical group was observed at this time point post-rupture^25^). The sample size was increased on the data monitoring and ethics committees’ advice, based on a prespecified masked review of the original sample size assumptions (primary outcome standard deviation).^15^ ^16^ An initial review on the overall patient population (not separated by treatment arm) was undertaken after the first 27 participants reached 24 week follow-up, at which time the standard deviation was lower than assumed. The standard deviation was reassessed when 75 patients had reached 24 week follow-up. At this point, the standard deviation was greater than the original sample size assumption. The sample size was therefore recalculated and the recommendation agreed with the independent trial steering committee was to increase the final sample size to 230 (115 per arm), which provided 80% power to detect a standardised difference of 0.5 in the work limb symmetry index (as measured through the heel rise endurance test) at 24 weeks after treatment, with 5% (two sided) significance and allowing 20% loss to follow-up.

### Statistical analysis

All participants were included in descriptive analyses. The baseline comparability of participant level data for each of the treatment groups was summarised and treatment compliance explored. Data distributions were explored visually using histograms and assessments for normality were carried out using percentile-percentile plots, quantile-quantile plots, and the Shapiro Wilk test. Normally distributed data were summarised as means and standard deviations, non-normally distributed data as medians and interquartile ranges, and categorical variables as frequency and percentages.

Primary and secondary outcome analyses were undertaken on modified intention-to-treat populations: all randomised participants with available outcome data were analysed in the groups to which they were allocated. Multivariable linear regression was used to investigate the effect of platelet rich plasma on Achilles tendon rupture recovery, adjusting for stratification variables and predefined prognostic variables (sex, body mass index, and smoking status) selected based on previous literature. Following a request from the data monitoring and trial steering committees, a post hoc analysis was undertaken that additionally adjusted for time from injury to injection, because participants could have had the injury up to 12 days before randomisation. Missing data and sensitivity analyses are described in the supplementary file.

We assessed data quality and the effect of the treatment received using complier average causal effect instead of the planned per protocol analysis. Complier average causal effect compares the average outcome of compliers in the treatment group with the average outcome of potential treatment compliers in the placebo group. Unlike a per protocol analysis, this analysis ensures that patients who did not receive the treatment allocated remain within the analysis, allowing for balance of randomisation factors to remain. We considered non-compliers to be participants from the platelet rich plasma group not receiving the allocated intervention or who received prepared platelet rich plasma that did not concentrate platelets compared with their whole blood. The analysis was repeated for the secondary outcome measures from the heel rise endurance test (limb symmetry index of heel rise repetitions and maximum heel rise height). 

We analysed the patient reported outcome measures using repeated measures, mixed effects, linear regression models, adjusting for stratification variables and predefined prognostic variables. Time between the intervention and outcome measurement was included in these models as a random effect factor. A P value less than 0.05 was considered indicative of a statistically significant difference in all analyses.

Complications were categorised as serious adverse events, or foreseeable or unforeseeable adverse events. We assessed masking success using the James and Bang indices^26^ ^27^; participants were asked after their heel rise endurance test at 24 weeks which treatment they believed they had received. The correlations between the primary outcome and both key blood parameters and platelet properties were explored visually and assessed using Pearson’s correlation. Analyses were conducted by Stata version 15.0 (StataCorp, College Station, TX). The trial is reported following the CONSORT statement and its related extensions.^28^

### Patient and public involvement

Our study protocol was developed following a pilot study that engaged with a panel of 75 patient and public involvement (PPI) representatives through a survey about key aspects of the proposed design. Subsequently, we reduced the number of follow-up contacts with participants. The trial steering committee had a PPI representative throughout all phases of set-up through to analysis. This PPI representative was involved in reviewing patient facing materials, including participant questionnaires and a presentation graphic to be used in future public engagement meetings. The results will be disseminated to trial participants, institutional public engagement meetings, and more widely through engagement with musculoskeletal charities and established national PPI networks.

## Results

**Fig 2:**
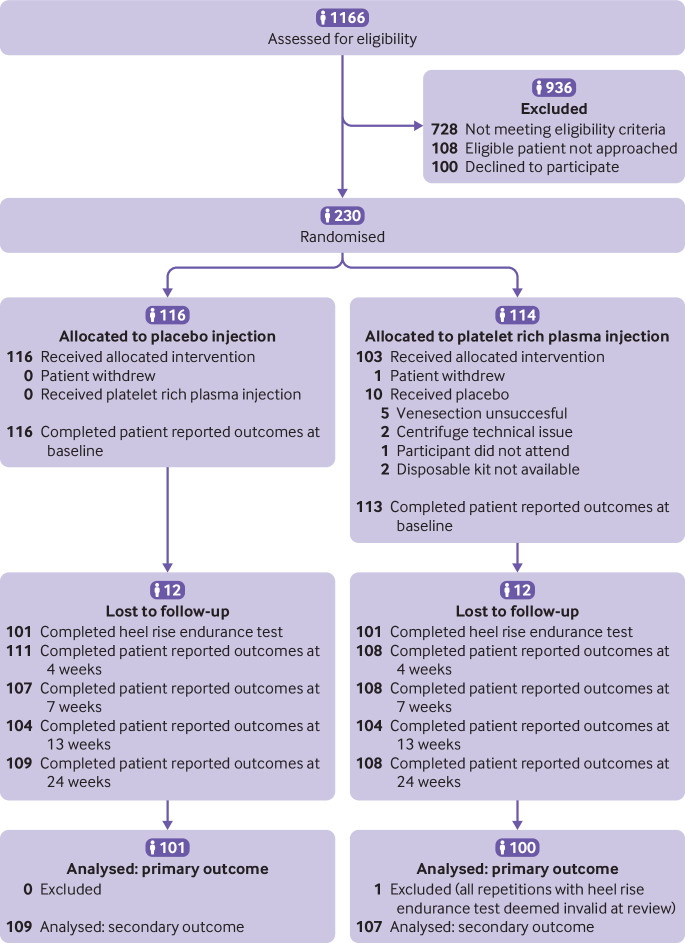
CONSORT diagram

Recruitment took place from 28 July 2015 to 18 September 2017, with follow-up completed on 9 March 2018. Of 1166 patients assessed for eligibility, 230 consented to randomisation (fig 2 and supplementary table S1). Baseline characteristics were well matched between the randomised groups, one participant in the platelet rich plasma group withdrew before completing the baseline questionnaire (table 1). Participants were on average 46 years old and 57 (25%) were female. Missing data accounted for 12% (28/230) of the primary outcome data and 6% (14/230) of the patient reported outcome data. We analysed 87% (201/230) of participants in the primary analysis. One participant’s data were excluded owing to invalid measurements.

**Table 1:** Baseline demographic and clinical characteristics of randomised participants, summarised by treatment group

Characteristic	Platelet rich plasma (n=113)	Placebo (n=116)
Age (mean (SD))	45.90 (13.74)	45.16 (12.43)
Female (No (%))	25 (22.12)	32 (27.59)
Body mass index (mean (SD))*	27.69 (5.29)	27.25 (4.22)
Time since injury (days; mean (SD))	5.35 (2.95)	5.20 (3.08)
Injured during sports participation (No (%))	81 (71.68)	76 (65.52)
Alcohol consumption (units/week; median (IQR))	6 (2-12)	8 (2-18)
Smoker (No (%))	14 (12.39)	13 (11.21)
Achilles tendon rupture score (median (IQR))†	12 (4, 19)	10 (4.5, 16)
Achilles tendon rupture score—functional limitation due to pain (median (IQR))‡	3 (0-7)	3 (0-5)
Pain visual analogue score (median (IQR))§	34 (9-63)	21.50 (9-54)
Patient specific functional score (median (IQR))¶	3 (0.67-6.67)	3 (1-6.67)
Short form 12 version 2® (median (IQR))**		
Pre-injury physical component	57.01 (50.11-58.05)	57.21 (48.33-58.43)
Pre-injury mental component	56.38 (50.48-59.05)	56.38 (49.30-59.21)
Post-injury physical component	30.09 (26.10-34.61)	28.84 (24.40-34.25)
Post-injury mental component	48.12 (37.75-59.06)	50.55 (41.58-58.85)
No (%) of comorbidities		
0	44 (38.94)	56 (48.28)
1	36 (31.86)	28 (24.14)
2	20 (17.70)	23 (19.83)
>2	13 (11.50)	9 (7.76)
No (%) of drugs affecting platelet function††	7 (6.19)	8 (6.90)
Whole blood analysis (mean (SD))‡‡		
Erythrocytes (×10^12^/L)	4.83 (0.59)	4.83 (0.48)
Leucocytes (×10^9^/L)	6.74 (2.05)	7.00 (1.90)
Platelets (×10^9^/L)	208.18 (77.73)	227.23 (65.54)

SD=standard deviation; IQR=interquartile range. One participant in the platelet rich plasma group withdrew before completing the baseline questionnaire.

* Data were not available for two participants in the placebo group.

† Scores were from 0 to 100, with 0 indicating major limitations and 100 indicating no limitations.

‡ Scores were from 0 to 10, with 0 indicating major limitations and 10 indicating no limitations.

§ Scores were from 0 to 100, with 0 indicating no pain and 100 indicating worst pain imaginable. Data were available for 93 participants in the platelet rich plasma group and 86 in the placebo group.

¶ Scores were from 0 to 10, with 0 indicating participants were unable to perform and 10 indicating they were able to perform at the prior level. Data on the patient specific functional scale were not available for one participant in the placebo group.

** Scores were from 0 to 100, with higher scores indicating better quality of life.

†† Question on drug treatment added during trial recruitment; data available for 81 participants in the platelet rich plasma group and 76 in the placebo group.

‡‡ Erythrocyte and leucocyte analyses available for 107 participants in the platelet rich plasma group and 114 in the placebo group; platelet analyses available for 104 participants in the platelet rich plasma group and 110 in the placebo group.

All of the placebo group (116/116) received their allocated treatment. In the platelet rich plasma group, one participant withdrew before receiving treatment, and 10 (9%) received a placebo injection instead of their allocated injection of platelet rich plasma because of a technical failure in the delivery of platelet rich plasma (fig 2). A consultant surgeon delivered the injections for 86 (76%) of the platelet rich plasma group and 87 (75%) of the placebo group. Surgical registrars or fellows or specialist physiotherapists delivered the remaining injections. Injections were delivered on average 5.3 (standard deviation 3.0) days after injury.

The prepared platelet rich plasma had 4.1-fold (95% confidence interval 3.6 to 4.5) greater platelet concentrations and 2.2-fold (95% CI 2.0 to 2.5) greater leucocyte concentrations than whole blood. Platelet quality measurements showed that platelet rich plasma was not activated before injection (CD62p expression 4.3%, standard deviation 5.0%) and was functional in vitro (60.1%, 22.3%). Growth factor concentrations were: vascular endothelial growth factor 1.0 ng/mL (standard deviation 0.7), transforming growth factor β1 131.9 ng/mL (74.4), platelet derived growth factor-AB 55.3 ng/mL (27.6), insulin-like growth factor 1 78.2 ng/mL (23.2), and fibroblast growth factor-basic 111.0 pg/mL (77.0).

Venous thromboembolism prophylaxis complied with individual hospital protocols and was prescribed to half of the participants (platelet rich plasma, 56/113; placebo, 58/116). The two groups had similar number of days to injured limb weight bearing (platelet rich plasma, mean 27 (standard deviation 24); placebo, 28 (16)) and starting ankle motion exercise (47 (20); 48 (20)).

We saw no evidence of a difference in muscle tendon function between the two groups, measured by the maximum work limb symmetry index (adjusted mean difference −3.9% (95% confidence interval −10.5% to 2.7%; platelet rich plasma, mean 34.7% (standard deviation 17.7%); placebo, 38.5% (22.8%); table 2). Sensitivity analyses supported the primary analysis findings, and results from the analysis for complier average causal effect were consistent with these conclusions (adjusted mean difference −4.3% (95% confidence interval −11.0% to 2.4%), supplementary table S2). A post hoc analysis was performed where the primary adjusted analysis was also adjusted for time from injury to injection. The results for limb symmetry index were consistent (adjusted mean difference −3.7% (95% confidence interval −10.2% to 2.8%); platelet rich plasma, mean 34.9% (standard deviation 16.7%); placebo, 38.6% (23.5%)). The James and Bang blinding indices indicated no evidence to suggest that masking was unsuccessful (supplementary table S3). Neither cellular and growth factor concentrations in platelet rich plasma nor quality measurements correlated with work limb symmetry index except for vascular endothelial growth factor (r=−0.23, P=0.03; table 3).

**Table 2:** Primary and secondary outcomes at 7, 14, and 24 week follow-up

Measure and follow-up	Platelet rich plasma			Placebo		Treatment comparison (adjusted difference (95% CI))*	P value
	No	Mean (SD)		No	Mean (SD)		
**Heel rise endurance test (%; mean (SD))†**							
Work limb symmetry index							
24 weeks	100	34.67 (17.66)		101	38.54 (22.82)	−3.87 (−10.45 to 2.71)	0.23
Maximum heel rise height, limb symmetry index							
24 weeks	100	55.10 (17.36)		101	55.43 (27.83)	−0.35 (−6.09 to 5.38)	0.90
Maximum heel rise repetitions, limb symmetry index							
24 weeks	100	50.08 (30.03)		101	60.75 (37.68)	−10.67 (−21.91 to 0.56)	0.06
**Achilles tendon rupture score**							
Mean (SD))‡							
4 weeks	107	28.46 (16.76)		109	30.61 (16.23)	−2.15 (−6.55 to 2.25)	0.34
7 weeks	107	37.58 (16.61)		109	38.62 (16.42)	−1.04 (−5.45 to 3.37)	0.64
13 weeks	107	51.66 (16.79)		109	53.11 (16.51)	−1.45 (−5.89 to 2.99)	0.52
24 weeks	107	64.99 (16.48)		109	65.53 (16.17)	−0.54 (−4.90 to 3.81)	0.81
Pain component score (mean (SD))§							
4 weeks	107	6.26 (2.98)		109	6.28 (2.89)	−0.02 (−0.81 to 0.76)	0.95
7 weeks	107	7.01 (2.96)		109	6.68 (2.93)	0.33 (−0.46 to 1.11)	0.42
13 weeks	107	7.49 (3.00)		109	7.24 (2.95)	0.25 (−0.54 to 1.05)	0.53
24 weeks	107	7.66 (2.93)		109	7.45 (2.88)	0.21 (−0.56 to 0.99)	0.59
**Patient specific functional score (mean (SD))¶**							
4 weeks	107	2.02 (2.18)		109	2.03 (2.12)	−0.01 (−0.58 to 0.56)	0.98
7 weeks	107	3.13 (2.17)		109	3.36 (2.14)	−0.23 (−0.80 to 0.35)	0.44
13 weeks	107	5.81 (2.19)		109	5.78 (2.15)	0.03 (−0.55 to 0.61)	0.91
24 weeks	107	7.20 (2.16)		109	7.49 (2.12)	−0.30 (−0.87 to 0.27)	0.31
**Short form 12 version 2**® (mean (SD))**							
Physical component							
4 weeks	105	38.87 (7.78)		108	39.00 (7.62)	−0.14 (−2.21 to 1.93)	0.90
7 weeks	105	40.73 (7.75)		108	42.42 (7.69)	−1.69 (−3.77 to 0.39)	0.11
13 weeks	105	45.76 (7.84)		108	46.27 (7.74)	−0.51 (−2.61 to 1.58)	0.63
24 weeks	105	50.24 (7.78)		108	49.44 (7.64)	0.80 (−1.27 to 2.88)	0.45
Mental component							
4 weeks	105	48.29 (9.55)		108	50.69 (9.34)	−2.40 (−4.94 to 0.14)	0.06
7 weeks	105	52.05 (9.51)		108	53.42 (9.44)	−1.37 (−3.91 to 1.18)	0.29
13 weeks	105	56.42 (9.60)		108	55.67 (9.48)	0.74 (−1.82 to 3.30)	0.57
24 weeks	105	53.79 (9.49)		108	55.60 (9.32)	−2.71 (−5.24 to −0.19)	0.04
**Pain visual analogue score (mean (SD))††**							
14 days	93	9.55 (21.45)		87	13.57 (21.51)	−4.02 (−10.30 to 2.26)	0.21

SD=standard deviation.

* Differences adjusted for age category (<55 *v* ≥55 years) and clustered by study site.

† Scores were injured/uninjured value×100, with 0 indicating no symmetry and 100 indicating perfect symmetry between limbs.

‡ Scores were from 0 to 100, with 0 indicating major limitations and 100 indicating no limitations.

§ Scores were from 0 to 10, with 0 indicating major limitations and 10 indicating no limitations.

¶ Scores were from 0 to 10, with 0 indicating participants were unable to perform and 10 indicating they were able to perform at the prior level.

** Scores were from 0 to 100, with 0 indicating worst and 100 indicating best. Differences further adjusted to account for participants pre-injury score.

†† Scores were from 0 to 100, with 0 indicating no pain and 100 indicating participants’ perceived worst pain imaginable.

**Table 3:** Correlation assessment between primary outcome and key blood parameters and platelet and growth factor properties in samples of platelet rich plasma

Key blood parameters and platelet properties	No	r	Variance (%)*	P value
**Blood cell counts**				
Erythrocyte count	91	0.13	1.59	0.23
Leucocyte count	91	−0.10	1.05	0.33
Platelet count†	88	0.13	1.65	0.23
**Platelet quality (resting)**				
Resting CD62p expression (%)	93	0.10	0.92	0.75
Mean fluorescence intensity	94	−0.03	0.07	0.97
**Platelet quality (activated)**				
Activated CD62p expression (%)	92	0.04	0.14	0.72
Mean fluorescence intensity	92	<−0.01	<0.01	0.97
**Growth factors**				
Insulin-like growth factor 1	93	0.12	1.25	0.29
Transforming growth factor β1	88	0.01	<0.01	0.96
Platelet derived growth factor-AB	90	<0.01	<0.01	1.00
Vascular endothelial growth factor	93	−0.23	5.35	0.03
Fibroblast growth factor-basic	93	−0.10	1.05	0.33

CD62p=P selectin.

* Proportion of variance in work limb symmetry index explained by blood parameter.

† As fluorescent platelet count.

**Fig 3:**
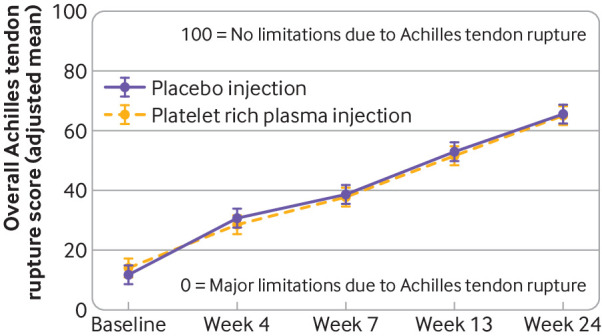
Results from repeated measures, mixed effects regression model, showing change in Achilles tendon rupture score in study participants (receiving platelet rich plasma *v* placebo) over time. Bars=95% confidence intervals

We saw no evidence of any differences between the platelet rich plasma and placebo groups in the other measures related to the heel rise endurance test (maximum heel rise height and heel rise repetitions), the patient reported outcomes of the Achilles tendon rupture score (fig 3), Achilles tendon rupture pain score, patient specific functional scale, or short form 12v2® at 4, 7, 13, or 24-week follow-up (table 2 and supplementary figs S1 to S3), or pain during the two weeks after injection (table 2 and supplementary fig S4). The two groups had similar adverse event rates related to their Achilles rupture or injection (platelet rich plasma, 84/113 (74%); placebo, 90/116 (78%) participants reporting at least one complication related to their Achilles rupture or injection; table 4). Re-rupture rates were 5% (6/113) in the platelet rich plasma group and 3% (4/116) for the placebo group, of whom nine participants went on to have surgical treatment. Rates of deep vein thrombosis were 5% (6/113) in the platelet rich plasma group and 4% (5/116) in the placebo group. One serious adverse event, an ST elevation myocardial infarction, occurred 2.5 hours after platelet rich plasma injection, which was deemed indirectly plausible.

**Table 4:** Treatment related adverse events during study. Data are the number of participants (%) experiencing event

	Platelet rich plasma group (n=113)	Placebo (n=116)
Participants experiencing at least one adverse event of any type*	84 (74.34)	90 (77.59)
Serious adverse events*	1 (0.88)	0
ST elevation myocardial infarction	1 (0.88)	0
Adverse events*	84 (74.34)	90 (77.59)
Foreseeable adverse events*	83 (73.45)	87 (75.00)
Mild discomfort or minor bleeding following injection	22 (19.47)	8 (6.90)
Technical complications of lower leg casting and splinting	38 (33.63)	28 (24.14)
Consequences of depending on walking aids	1 (0.88)	1 (0.86)
Syncopal episode related to venesection or tendon injection	0	0
Discomfort at rupture site during rehabilitation	9 (7.96)	10 (8.62)
Swelling or bruising of the lower leg and foot	68 (60.18)	77 (66.38)
Deep vein thrombosis in a lower limb	6 (5.31)	5 (4.31)
Re-rupture of treated Achilles tendon	6 (5.31)	4 (3.45)
Unforeseeable adverse events*	16 (14.16)	19 (16.38)
Serious infection of injection site of Achilles tendon rupture	0	0
Skin breakdown or ulceration of treated lower leg	13 (11.50)	13 (11.21)
Severe pain (more than simple analgesia) >10 days after injection	6 (5.31)	6 (5.17)
Other adverse events related to treatment or Achilles tendon rupture*	13 (11.50)	13 (11.21)
Frequent discomfort at injection site	5 (4.42)	6 (5.17)
Infection at injection site confirmed by doctor	0	3 (2.59)
Infection at non-injection site†	0	3 (2.59)
Other problem‡	9 (7.96)	6 (5.17)

* Participants could have experienced multiple adverse events so number of participants reporting foreseeable and unforeseeable adverse events may not add up to the overall total reporting.

† Infections included cellulitis, pneumonia, and an infected insect bite on the treated leg.

‡ Participant specific complications associated with treatment or rupture not covered by other complication types.

## Discussion

### Principal findings

This large randomised controlled trial found no evidence that platelet rich plasma injections improved muscle tendon function, patient reported function, pain, goal attainment, or quality of life in patients with acute Achilles tendon rupture. The hypothesised benefits of platelet rich plasma in tendon injury healing, based on encouraging findings in laboratory studies, did not translate into a detectable patient benefit. Our use of a standardised device for platelet rich plasma preparation, quality control procedures across 19 hospitals, and robust trial design and conduct strengthen the confidence in our findings.

We used outcomes based on the main limitations people experience after an acute tendon rupture. Muscle tendon function was selected as the primary outcome measure because this is the primary impairment after Achilles tendon rupture and can be quantitatively assessed. We also used the validated patient reported outcome (Achilles tendon rupture score),^29^ because we recognised that platelet rich plasma could have wider effects on patients’ recovery experience. The consistency and precision in the estimates indicated no evidence of platelet rich plasma efficacy in any of the assessed outcomes. The finding of the PATH-2 trial highlights that the use of platelet rich plasma preparations in soft tissue injuries must be questioned unless supported by robust evidence indicating positive outcomes.

### Comparison with other studies

Systematic reviews have synthesised evidence from 37 clinical trials on platelet rich plasma applications in musculoskeletal injuries so far.^10^ ^11^ Comparisons with our study are challenging owing to heterogeneity in clinical applications, lack of standardisation of platelet rich plasma preparations and quality controls, underpowered studies, and potential confounders such as concurrent surgery in those trials. Achilles ruptures are increasingly being treated non-surgically,^30^ but previous trials of platelet rich plasma in Achilles tendon rupture have used platelet rich plasma as an adjunct to surgical repair, and have low statistical power and high loss to follow-up.^12^ ^13^

### Strengths and limitations of the study

To our knowledge, PATH-2 was the largest trial so far to investigate the efficacy of platelet rich plasma in acute tendon ruptures. The interventions were embedded into usual care pathways, which enhances the generalisability of the findings. Platelet rich plasma analysis indicated that our preparation method produced a leucocyte and platelet rich plasma of optimal quality that would have provided a supraphysiological concentration of leucocytes and platelets capable of degranulating and releasing high concentrations of growth factors on injection, with few exceptions.

Platelet rich plasma preparations are often poorly reported and not standardised between trials, leading to heterogeneity and uncertainty in the literature.^31^ These preparations could contain supraphysiological or subphysiological concentrations of leucocytes. The effects of leucocytes in platelet rich plasma for tendon healing are uncertain, with in vitro investigations identifying both potential benefits and limitations at the cellular level.^32^ Currently, leucocyte rich preparations are commonplace in clinical practice, as buffy-coat-derived platelet rich plasma results in substantial leucocyte and red cell inclusion. In PATH-2, we used a method to prepare leucocyte and platelet rich plasma that had cellular and growth factor levels consistent with the preparation device manufacturer’s specifications^33^ and those observed within other controlled laboratory studies of healthy individuals.^34^ ^35^ ^36^ Unlike many previous trials,^37^ we fully defined what the participants received and importantly found no correlation between any platelet or leucocyte parameters of platelet rich plasma and muscle tendon function outcome. We also did not see a correlation between growth factors and muscle tendon function outcome, except for a weak negative correlation with vascular endothelial growth factor. The analysis on complier average causal effect had similar findings to the primary analysis, indicating that observed deficiencies in adhering to the intervention protocol did not affect the final results.

The PATH-2 trial had some limitations. Different volumes of whole blood were taken from the two randomisation groups (55 mL platelet rich plasma *v* 5 mL placebo). Despite safeguards, participant masking could have been compromised. However, indices used to measure participant masking after assessment of the primary outcome indicated that participants did not accurately predict their allocated treatment. Although the rehabilitation protocol set boundaries on the length of immobilisation and weight bearing, variation is still possible in these factors and in the content of the physiotherapy sessions. However, time to weight bearing, duration of immobilisation, and referral rates to physiotherapy were balanced between the groups, and centre effects were managed with stratification and adjustment of estimates.

### Conclusions and policy implications

We found no evidence that, compared with placebo, platelet rich plasma injections have an effect on objective muscle tendon function, patient reported function, or quality of life after acute Achilles tendon rupture, indicating that platelet rich plasma offers no patient benefit.

**What is already known on this topic**

After Achilles tendon rupture, patients experience limitations in activities that load the tendon, resulting in work incapacity and many months off sport and work; accelerating recovery and improving quality of the tissue repair is therefore desirable
Autologous platelet rich plasma containing supraphysiological platelet concentrations from whole blood is used extensively in musculoskeletal medicine
Although platelet rich plasma positively affects cellular and physiological tendon healing under laboratory conditions, quality of clinical efficacy evidence is poor

**What this study adds**

There is no evidence to indicate that injections of platelet rich plasma improve objective muscle tendon function, patient reported function, or quality of life after acute Achilles tendon rupture compared with placebo, or that they offer any patient benefit

## Web extra

Extra material supplied by authors

**Web appendix:** Supplementary file

## Notes

Contributors: DJK and JA contributed equally. JA, DJK, PHa, PHu, SJD, SEL, and KW contributed to the study concept and design. All the authors contributed to the acquisition, analysis, and interpretation of data. DJK, HMO’C, SJD, and KW drafted the manuscript. All the authors critically revised the manuscript for important intellectual content. HMO’C, MMS, SJD, SEL, and KW conducted the statistical analysis. DJK, JA, PHa, PHu, SJD, SEL, and KW obtained funding. SW, SRP, and JYT provided administrative and technical support. SEL and KW supervised the study. The corresponding author attests that all listed authors meet authorship criteria and that no others meeting the criteria have been omitted. DJK and KW act as guarantors and take responsibility for the integrity of the data and the accuracy of the data analysis.

Ethical approval: The study was approved by the National Research Ethics Service Oxfordshire Committee A (reference 14/SC/1333) and overseen by an independent trial steering committee and data monitoring and ethics committee. We attest that we have obtained appropriate permissions and paid any required fees for use of copyright protected materials.

Data sharing: Anonymised datasets can be made available on reasonable request after approval from the trial management committee and after signing a data access agreement. Proposals should be directed to the corresponding author.

The lead author affirms that the manuscript is an honest, accurate, and transparent account of the study being reported; that no important aspects of the study have been omitted; and that any discrepancies from the study as planned and registered have been explained.
