# Supplementary material for: Platelet rich plasma injection for acute Achilles tendon rupture: PATH-2 randomised, placebo controlled, superiority trial
Source: BMJ. 2019 Nov 20;367:l6132. doi: 10.1136/bmj.l6132 (PMC6863552; doi:10.1136/bmj.l6132)
Supplement: Supplementary file 1 — Web appendix: Supplementary file [file keed051671.ww.pdf]

Supplementary File

Table of Contents

Summary of amendments to the protocol..........2

Amendment to eligibility criteria..........3

Heel rise endurance test..........5

Blood and platelet rich plasma sample handling and preparation..........7

Missing data - sensitivity analyses..........9

Table S1: Reasons for not meeting eligibility criteria..........11

Figure S1: Results from the repeated measures mixed effects regression model demonstrating the change in Achilles Tendon Rupture Score (ATRS) pain measure in PRP injection and placebo injection participants over time...........12

Figure S2: Results from the repeated measures mixed effects regression model demonstrating the change in Patient-Specific Functional Score (PSFS) in PRP injection and placebo injection participants over time...........13

*Figure S3: Results from the repeated measures mixed effects regression model demonstrating the change in (a) SF-12® v2 Physical Component Score (PCS) and (b) Mental Component Score (MCS) in PRP injection and placebo injection patients over time, with mean pre-injury score presented.**..........14

Figure S4: Results from the repeated measures mixed effects regression model demonstrating the change in pain as reported by the Visual Analogue Score (VAS) in PRP injection and placebo injection participants over time...........15

Table S2: Primary outcome sensitivity analyses..........16

Table S3: James and Bang blinding index results, including participants reporting 'don't know' for treatment allocation..........17

Summary of amendments to the protocol

Main protocol amendments after the initial version approved are outlined below:

- February 2015, protocol version 2.0: trial registration added and blood handling training materials clarified
- March 2016, protocol version 3.0:
  - addition of extended scope physiotherapist to the clinical staff involved in assessing and treating Achilles tendon ruptures and conducting injection under the supervision of the orthopaedic surgeon, to reflect current NHS practice in some NHS Trusts.
  - changes to eligibility criteria: see following section for full details.
  - change to randomisation process to correct error in randomisation system: from randomisation with stratification by strata (centre and age group) to using minimisation, a dynamic computer generated allocation system based on the two strata. The change to randomisation was required due to imbalance in participants' age group stratum following a systems issue. The underlying systems issue was fixed and a change to the randomisation strategy was implemented to avoid this imbalance being preserved throughout the study. The randomisations allocated prior to the change were not altered. This approach was reviewed and approved by the Sponsor, DMSC, TSC and the Ethics Committee.
  - Collection of current medications data at baseline.
  - Inclusion in protocol of questions asked at 24 weeks on participant's experience of trial.
  - Clarification on excess treatment costs for study specific consumables.
  - DVT and re-rupture changed from "unforeseeable" to "foreseeable" adverse events.
- March 2016, version 4.0: Removal of "draft" from filenames, watermark and tracked changes from documents for version 3.0.

- April 2017, version 5.0: inclusion of 2-year extended follow-up. Dates changed to include 9-month extension, as agreed with funder.
- July 2017, version 6.0: sample size changed from 214 to 230 after blinded review of variability in primary outcome data (see main manuscript for details). Dates changed to include 2-month extension, as agreed with funder.

Amendment to eligibility criteria

In May 2016 (nine months in to recruitment) a substantial amendment was implemented following approvals that included changes to the eligibility criteria. The changes and rationale are summarised below:

- For eligibility for the PATH-2 trial, Achilles tendon rupture needed to be complete, partial ruptures were not eligible. We clarified this in the protocol based on questions from sites to ensure the target population was included in the study
- We clarified that Achilles tendon rupture at the insertion to the calcaneum (bone attachment) and the calf muscle (musculotendinous junction) were to be excluded.
- The original eligibility criteria of being within seven days from injury proved to be controversial and difficult to implement. Although being close to the date of injury remained desirable, we extended this to within 12 days for following reasons:
    - Treatment within seven days was proving to be incompatible with the treatment pathway for many acute Achilles tendon rupture in the NHS hospitals taking part in the trial
    - Most patients were arriving at orthopaedic clinics within 12 days for acute treatment so many patients in the target population (acute ruptures) were not being offered entry to the study. This had serious implications for the generalisability of the research

    - Treatment within 12 days was still deemed within the acute phase of the injury and there is no strong evidence that the effects of Platelet Rich Plasma (PRP) would have different effects if administered a few days later. It also reflected the period within which non-surgical management is usually preferred in the NHS. Later delayed presentations often get selected to surgical treatment

- The upper age limit of 70 years was removed to ensure we are opening the opportunity to participate to the wider population to whom the research was relevant. The age criteria was changed to 18 years or over and concurrently we added a second criterion to ensure we are including individuals who were ambulatory without the use of walking aids or assistance of another person. We decided to focus the study on individuals who were independent with ambulatory activities such as walking and stair climbing as those with very limited or no ambulatory function were not likely to benefit from the intervention under investigation. There was no strong evidence that those over 70 would respond differently to the PRP intervention and they are in fact more likely to be managed non-surgically, our target population. It is increasingly recognised that higher level physical activity and sports participation in older age is increasing, so Achilles ruptures are an important injury for adults of all ages.
- There are different uses of anticoagulant and these are reflected in their dose. We clarified that the exclusion criteria related to anticoagulant use related to a higher treatment dose, not the lower preventative doses commonly used in this patient group to prevent deep vein thrombosis in the early phase of being immobilised in a cast or brace.

Heel rise endurance test

The heel rise endurance test (HRET) was preceded by the participant watching a video demonstration of the HRET and reading standardised written instructions detailing the test. A warm-up followed, which involved five minutes of usual pace walking, then 10 double-leg heel rises. Before testing each leg, participants were asked to stand on an incline box.

The HRET was performed on the uninjured limb, then the injured leg. Testing started when the participant was in position, standing on one leg on the 10° incline box (so the ankle was in a dorsiflexed position) with the cord from the linear encoder strapped to the heel (Figure 1).

The following standardisation was applied:

- ankle starting position of 10° dorsiflexion produced by conducting the HRET on a custom-made 10° incline box
- knee in full extension
- height of each repetition as high as possible
- pace of 30 rises per minute guided by a digital metronome
- balance support by the finger tips only

There were strictly defined test termination criteria: participants either stopped (i.e. volitional task failure) or were verbally instructed to stop with both feet flat on the box whenever any of the following test termination criteria were observed:

1) inability to keep pace with the metronome

2) inability to maintain full knee extension of the standing leg

3) using more than fingertip support

The desired endpoint was volitional task failure. However, the outcome assessors were encouraged to use verbal prompts whenever the termination criteria were observed and to stop the test if the participant did not respond to two consecutive prompts.

A member of the PATH-2 team gave face-to-face training and demonstration to each outcome assessor before their first participant was invited to their six-month follow-up. Assessor training material consisted of high quality training videos by the PATH-2 team produced by Oxford Medical Illustrations (Oxford University Hospitals NHS Foundation Trust) and a training and reference manual.

The linear encoder was sensitive so could record minimal movements that might not represent actual heel rises (e.g. participants tend to step off the box or lift the leg up at the end of the test). To dismiss erroneous recordings, two members of the study team (Chris Byrne, Jacqueline Thompson, Susan Wagland or David Keene) masked to treatment allocation independently reviewed videos of all assessments where participants consented to recording. The invalid heel rise repetitions in the HRET data were identified so that they could be dealt with in the analysis of missing data.

Blood and platelet rich plasma sample handling and preparation

Venous blood (1-5 ml) from all participants were anticoagulated within ethylenediaminetetraacetic acid vacutainers (Becton Dickinson, Plymouth, UK). The 50 ml venous blood for the PRP group was used for PRP preparation. 8 ml of sterile PRP was produced. 4 ml was used for injection into the Achilles tendon and the remaining 4 ml was divided into four 1 ml aliquots. One microtube was frozen at -70^0C for storage until the end of the trial for batch measurement of growth factor levels.

The frozen samples were sent to the central laboratory at the University of Birmingham on dry ice by courier. All samples arrived frozen and were batched into appropriate sizes for growth factor measurements. For analysis, frozen samples were all thawed at 37^0C for 10 minutes. 1/40th volume of 20% Triton-X-100 was then added to ensure full cell lysis prior to a further incubation at 37^0C for 10 minutes. Samples were then mixed and centrifuged at 1500 g for 10 minutes at room temperature to remove insoluble debris. Supernatants were then removed and assayed for 5 different growth factors (platelet derived growth factor-AB, insulin-like growth factor 1, vascular endothelial growth factor, fibroblast growth factor-basic (b) and transforming growth factor β1) with commercial ELISA kits (Biotechne, Abingdon, Oxfordshire, UK). Optimal sample dilutions for each growth factor were pre-determined by assaying PRP samples prepared from normal volunteers using the identical method of preparation. Diluted samples, blanks and standards were pipetted in duplicate into 96 well plates coated with capture antibodies and incubated as instructed within each growth factor ELISA kit. The measured values were comparable with previous papers detailing growth factor levels in PRP prepared by the same device that was used in the trial and known normal levels of growth factors in platelets.

Two of the three remaining PRP aliquots (each 1 ml) were stimulated at room temperature for 5 minutes by introducing them to two other tubes (Platelet Solutions Ltd, Nottingham, UK) containing either saline alone to provide an unstimulated baseline or adenosine diphosphate and U46619 to fully activate the platelets. These samples were then fixed using 1 ml PAMFix (Platelet Solutions Ltd, Nottingham, UK). The whole blood, unfixed PRP and these two tubes were transported at room temperature by courier to the Institute of Inflammation and Ageing at the University of Birmingham and processed on arrival.

Missing data - sensitivity analyses

Missing HRET data was defined and handled as follows:

- Participants with true missing data: Participants who did not complete their 24-week HRET assessment and participants who experienced technical errors during their assessment. These participants were included in the dataset with missing values.
- Participants with true zero measurements: Participants who attempted to complete their HRET assessment but their attempts were insufficient for the encoder to record any results. These participants were included in the dataset with zero values.
- Participants with potential zero measurements: Participants who attended their 24-week follow-up appointment but did not attempt their HRET assessment. These participants were included in the dataset with zero values.

The distribution of the data was nearly normal, with a small raise around zero due to the missing data processes followed above. The impact of zero inflation of the primary outcome measure was assessed using a Two-Part Model to compare participants with positive work LSI and those with zero measures, and to assess the effect of including those with zero measures on the primary outcome results.

Missing HRET data were handled in two ways. For patients missing data for individual heel rise repetitions, simple imputation of the median concentric displacement of their other repetitions for that leg was carried out. For patients missing data for entire HRET assessments, multiple imputation using the chained equations (MICE) procedure was used to create a series of complete datasets (observed + imputed) in which analyses were performed individually, with parameter estimates and standard errors combined using the Rubin's rule approach. The method utilised for parameter inclusion was to use the predictor variables

already in the model and to further account for any variables with an R-squared value of 0.2 or greater following correlation assessments. This resulted in three additional variables being selected for the primary outcome analysis: whether or not the participant jogged or ran, undertook weight training, or participated in squash before their injury.

Table S1: Reasons for not meeting eligibility criteria

**Reason for not meeting eligibility criteria**	**Number of times reason given**
Aged 17 or under	6
Aged 71 or over*	18
Not ambulatory prior to injury†	4
Suspected Achilles tendon rupture, but surgeon confirmed did not	172
Presented more than 7 days post-injury*	42
Presented more than 12 days post-injury†	157
For operative treatment	120
Not able or willing to participate in all study requirements	21
Not able to attend a study site for 24-week follow-up	6
Achilles tendon rupture at insertion or musculotendinous junction	56
Previous major tendon/ankle injury/deformity to either lower leg	83
History of diabetes mellitus	38
Known platelet disorder or hematological disorder	4
Current use of systemic cortisone or anticoagulant	10
Evidence of lower limb gangrene or peripheral vascular disease	3
History of hepatic or renal impairment or dialysis	6
Pregnant or breast feeding	2
Receiving or received radiation or chemotherapy within last 3 months	3
Had inadequate venous access for drawing blood	0
Other significant disease / disorder which could put participant at risk / influence results of study or patient's ability to participate in study	18
Reason not recorded	2
**Total**	**771‡**

* category not used after May 2016

† category implemented May 2016

‡ Some participants had more than one reason marked therefore total is greater that the number of participants not meeting eligibility criteria (n=728)

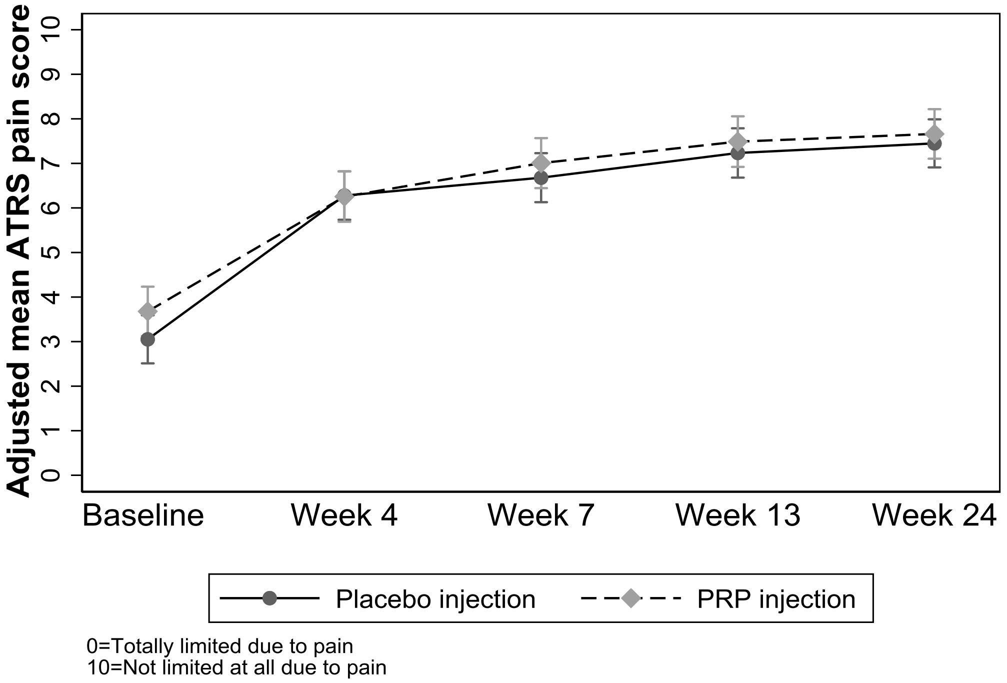

Figure S1: Results from the repeated measures mixed effects regression model demonstrating the change in Achilles Tendon Rupture Score (ATRS) pain measure in PRP injection and placebo injection participants over time.

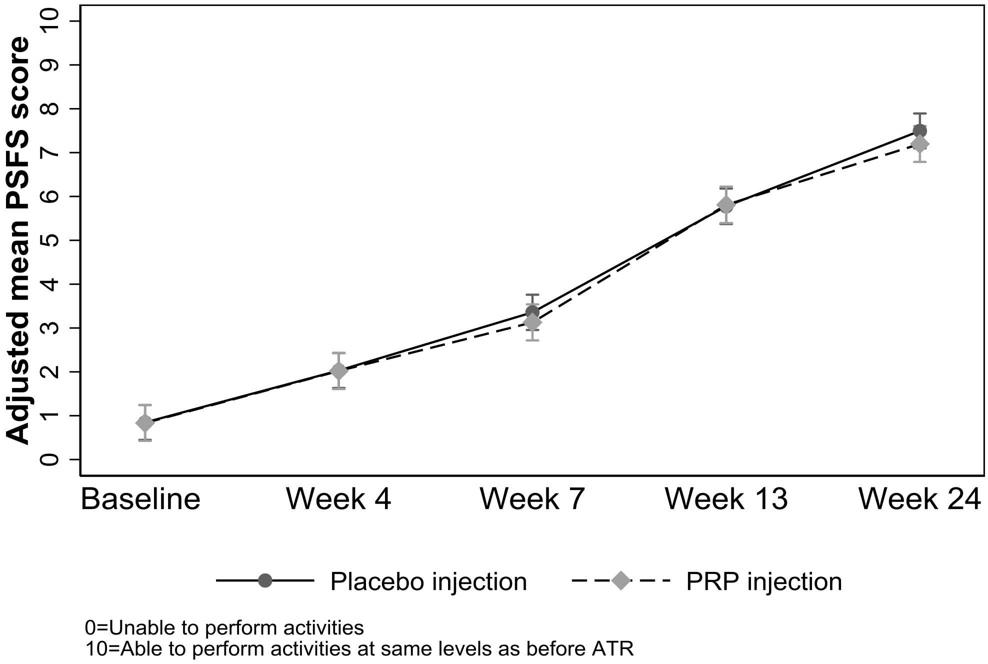

Figure S2: Results from the repeated measures mixed effects regression model demonstrating the change in Patient-Specific Functional Score (PSFS) in PRP injection and placebo injection participants over time.

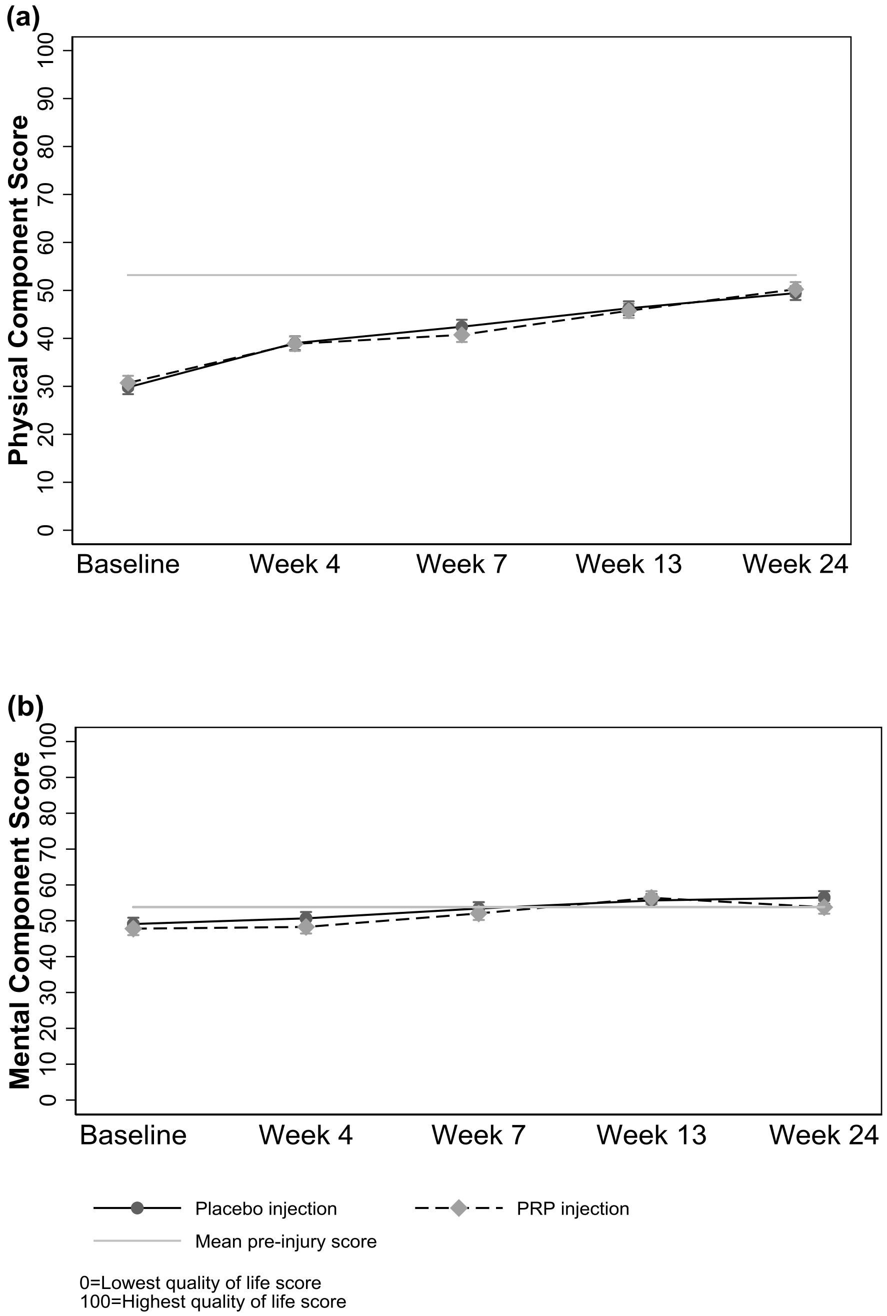

*At the baseline assessment participants were asked for pre-injury and current post-injury status.

Figure S3: Results from the repeated measures mixed effects regression model demonstrating the change in (a) SF-12® v2 Physical Component Score (PCS) and (b) Mental Component Score (MCS) in PRP injection and placebo injection patients over time, with mean pre-injury score presented.*

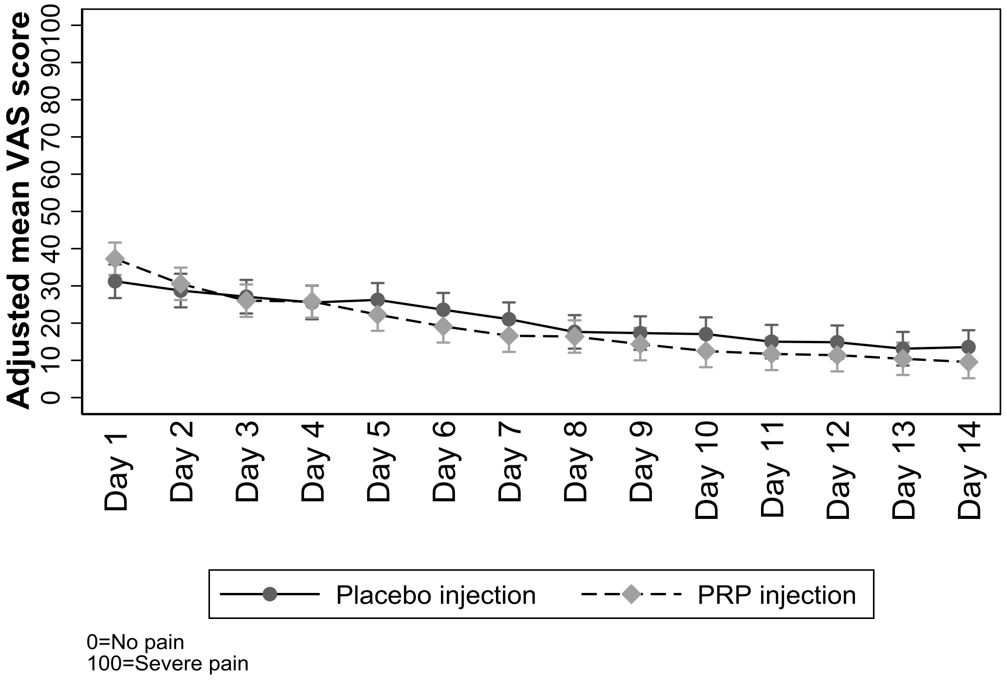

Figure S4: Results from the repeated measures mixed effects regression model demonstrating the change in pain as reported by the Visual Analogue Score (VAS) in PRP injection and placebo injection participants over time.

Primary outcome sensitivity analyses

Table S2: Primary outcome sensitivity analyses

	PRP injection		Placebo injection		Treatment comparison	*P*-value
Measure	n	Mean (SD)	n	Mean	Adjusted difference (95% CI)*	
**Work limb symmetry index -%†**						
ITT	100	34.67 (17.66)	101	38.54 (22.82)	-3.87 (-10.45 to 2.71)	0.23
Two-part model‡	83	41.46 (16.02)	87	45.04 (16.87)	-3.58 (-9.35 to 2.21)	0.21
Simple imputation	100	34.68 (17.68)	101	38.55 (22.78)	-3.87 (-10.46 to 2.71)	0.23
MICE	113	34.58 (22.25)	116	38.53 (25.73)	-3.94 (-11.12 to 3.23)	0.26
CACE	100	34.23 (20.37)	101	38.55 (22.08)	-4.31 (-11.00 to 2.38)	0.21
Adjusted for additional prognostic factors§	100	34.36 (18.59)	101	38.85 (22.34)	-4.49 (-11.34 to 2.36)	0.19

Abbreviations: CI, confidence interval; ITT, intention-to-treat; MICE, multiple imputation using the chained equations; CACE, complier average causal effect.

* Differences were adjusted for age category (<55 years, ≥55 years) and study site except where specified.

† Scores were injured/uninjured value x100, with 0 indicating "no symmetry" and 100 indicating "perfect symmetry" between limbs.

‡ Primary outcome data followed a near-normal distribution, with a small elevation around zero. LSI data did not require transformation prior to inclusion in the final model as post-estimation assessments indicated that the distribution was suitable for the regression technique employed. The impact of zero inflation of the primary outcome measure was assessed using a two-part model, to: i) identify differences between participants with positive work LSI results compared to those with zero measures by modelling the likelihood of a patient recording a non-zero LSI measurement, and ii) identify the impact of inclusion of participants with zero measures on the primary outcome results.

§ Difference additionally adjusted for Body Mass Index, sex, and smoking status.

Table S3: James and Bang blinding index results, including participants reporting ‘don’t know’ for treatment allocation

	Index	Standard error	95% confidence interval	
James*	0.82	0.03	0.77	0.86
Bang: PRP injection†	0.14	0.07	0.04	0.25
Bang: Placebo injection†	-0.18	0.06	-0.27	-0.09

* Index value greater than 0.5 indicates blinding likely to have been achieved

† Absolute index value less than 0.2 indicates blinding likely to have been achieved
